# Inclusion of Sub-Antarctic Macroalgae (*Gigartina skosttsbergii*) as Feed Ingredient for Grazing Sheep

**DOI:** 10.3390/ani15131976

**Published:** 2025-07-04

**Authors:** Cinthya Glucevic, Navid Ghavipanje, Lizbeth E. Robles-Jimenez, Sergio Radic-Schilling, Manuel Gonzalez Ronquillo

**Affiliations:** 1 Departamento de Ciencias Agropecuarias y Acuícolas, Universidad de Magallanes, Punta Arenas 6200000, Chile; cinthya.glucevic@umag.cl (C.G.); sergio.radic@umag.cl (S.R.-S.); 2 Department of Animal Science, Faculty of Agriculture, University of Birjand, Birjand 97175-331, Iran; navid.ghavipanje@gmail.com; 3 South Khorasan Agricultural and Natural Resources Research and Education Center, Agricultural Research, Education and Extension Organization (AREEO), Birjand 9735197311, Iran; 4 Laboratorio de Agroecología, Instituto de Investigaciones en Ecosistemas y Sustentabilidad (IIES), Universidad Nacional Autónoma de México, Morelia 58190, Michoacan, Mexico; lizroblez@hotmail.com; 5 Departamento de Nutricion Animal, Facultad de Medicina Veterinaria y Zootecnia, Universidad Autonoma del Estado de Mexico, Instituto Literario 100 Ote, Toluca 50000, State of Mexico, Mexico

**Keywords:** feed supplement, *Gigartina skottsbergii*, macroalgae, methane, sheep

## Abstract

Two experiments were carried out to evaluate the inclusion impacts of macroalgae *Gigartina skottsbergii* (Gs) for grazing sheep. Experiment (Exp.) 1 studied the effect of Gs on in vitro gas production (IVGP), dry matter (DM) digestibility (IVDMD), and methane (CH_4_) emission using three fistulated sheep and 96 h incubation of samples. In Exp. 2, ten Dohne Merino ewes [5-year-old; 47 ± 0.14 kg body weight (BW)] were randomly assigned to dietary treatments supplemented with Gs at 0 or 450 g DM/d per animal. The study lasted 31 days and was preceded by an adaptation period of 9 days. The BW, body conditional score (BCS), and blood were sampled at the first and the last day of the trial. The results of Exp. 1 showed that Gs supplementation reduced (MCP, *p* = 0.026) gas production (A), lag time (*p* = 0.013), and IVDMD (*p* = 0.071), while it enhanced partition factor (PF96; *p* = 0.004) and microbial crude protein (MCP) (*p* = 0.054). The concentration of CH_4_ decreased after 3 h (*p* = 0.0002), 6 h (*p* = 0.013), and 12 h (*p* = 0.010) with a tendency at 9 h (*p* = 0.109) and 24 h (*p* = 0.068). In Exp. 2, there were no diet effects on the initial BW (IBW, *p* = 0.77), final BW (FBW, *p* = 0.91), and average daily gain (ADG, *p* = 0.24) of ewes; however, Gs supplementation decreased BCS (*p* = 0.004). Of all blood parameters, only the concentration of glucose (*p* = 0.021) and albumin (*p* = 0.011) decreased in the Gs group. Overall, our results revealed that the dietary inclusion of Gs (at 450 g DM/d) affected neither the BW nor ADG of ewes; however, Gs was accompanied by lesser IVGP and CH_4_ emission.

## Simple Summary

Macroalgae have been incorporated into livestock diets as novel protein and carbohydrate sources with bioactive compounds that could benefit both human and animal health. Recent studies demonstrated that macroalgae can reduce methane production in ruminants without affecting performance, supporting their use as a promising feed ingredient. In this study, we first evaluated the in vitro gas kinetic and methane emission of *Gigartina skosttsbergii* (red macroalgae), and secondly, we used in vivo assessment to obtain the effects of dietary *Gigartina skottsbergii* (Gs) supplementation for grazing sheep on growth performance and blood parameters. Our results showed that the dietary inclusion of Gs (at 450 g DM/d) for sheep did not alter body weight, average daily gain, and most of the blood parameters. The implementation of Gs mitigated enteric methane (CH_4_) emission, in vitro, at this inclusion rate; however, this was accompanied by lower in vitro gas production (IVGP) and in vitro dry matter degradability (IVDMD). Further research is needed to refine the potential impacts of Gs on ruminal fermentation, methane emission, and product quality.

## 1. Introduction

The increasing demand for ruminant feed, affected by pandemics and climate change, necessitates the search for sustainable feed alternatives [1,2]. Macroalgae, also known as seaweed, comprise one of the prospective solutions as a promising alternative feed source for ruminants that are valued as a cost-effective and unconventional feed source over terrestrial plants [3]. This is attributed to their ability to utilize salt water instead of fresh water, minimize the need for industrial fertilization, and achieve superior biomass productivity per unit of surface area [4]. Some evidence has demonstrated that the incorporation of macroalgae contributes to the mitigation of the eco-environmental footprint of ruminant production without impairing animal health and productivity, although research is still in its infancy and continues to evolve [3,4,5]. In the last decade, the global cultivation of macroalgae has doubled, reaching an annual fresh weight of approximately 32 million tons [6]. It has been well established that macroalgae biomass is rich in crude protein, fatty acids, minerals, vitamins, and bioactive compounds that makes it a useful ingredient when conventional feedstuffs are of poor quality [4,7].

The Magallanes and Chilean Antarctic Region are characterized by extensive sheep farming [8], with the feeding mostly based on native grasslands made up of low-quality forage species, representing 96% of the total area used for grazing in the region [8]. The nutritional characteristics of the forages in these natural pastures are characterized by high dry matter (DM) and low crude protein (CP) content (10.5% in spring and 4.2% in winter, DM basis) [4,9], which do not meet the production requirements of sheep and make the use of supplementations inevitable. Chile has an extensive coastline that facilitates the utilization of marine feed ingredients such as macroalgae [10]. *Gigartina skottsbergii* (Gs) is one of the most important carrageenophytes in Chile, with production generated entirely from wild populations [6]; it represents an abundant, local feed source that does not compete with terrestrial feed source while showing nutritional potential as a ruminant feed [9].

Recently, the macroalgae-based animal feeding has been extensively reviewed [3,4,9,11,12]. There is also available information from both in vitro and in vivo experiments on the feasibility of different macroalgae for ruminants. It has been proposed that seaweed (*Ulva lactuca*) might be a suitable feed supplement for sheep [13]. Rjiba-Ktita et al. [7] reported that green algae can be included in the fattening lamb’s diet (up to 200 g/kg) as a replacement for protein supplements without negative impacts on intake and performance. It has been also shown that feeding red algae (*Lithothamnion calcareum*) to dairy cows [14] and brown algae (*Ascophyllum nodosum*) to lactating ewes [15] led to a higher milk production. The addition of 10 g/day (DM basis) of *Ascophyllum nodosum* has also has beneficial effects on forage digestibility in low-quality forage diets in steers [16]. It has been demonstrated in vitro that the addition of macroalgae (*A. taxiformis* and *Z. farlowii*) at a dose rate of 5% DM reduced methane production by up to 74% [17]. The integration of seaweeds into sheep rations [13] has been used in coastal areas in times of fodder shortage and has potential for use in ruminants [3,9]. However, an in vitro study [7] reported that the inclusion of aquatic plant species (*Ruppia maritima*, *Chaetomorpha linum*, and *Ulva lactuca*) should not exceed 200 g/kg in concentrate feeds, due to the reduction of the rate and extent of degradation. The large-scale application of macroalgae in ruminant diets faces specific challenges, including a high level of ash (~20–40% of DM), posing risks of mineral toxicity and low organic matter (OM) digestibility, which impairs the degradation of fiber and protein [18].

Despite the promising prospects of macroalgae-based ruminant feeding in terms of mitigation, food–feed–fuel competition, and methane emission, as well as producing functional foods [3,11], there is no study, to the best of our knowledge, regarding the use of Gs (red algae), endemic macroalgae to the Magallanes Region and Chilean Antarctica, in sheep feeding. Among the locally available macroalgae, red seaweed Gs is the only species harvested and commercially processed in the Magallanes Region. Its legal status allows for large-scale wild harvest and industrial dehydration, unlike brown macroalgae such as *Macrocystis pyrifera*, which are ecologically protected [19,20]. This regional specificity, combined with Gs’s nutritional potential and local availability through commercial processors in Punta Arenas, supports its selection as a practical and sustainable supplement for ruminant feeding [19,20]. Hence, the present investigation aimed, first, to explore the additive effects of Gs on in vitro fermentation parameters and methane (CH_4_) emission and, second, to evaluate the impacts of dietary Gs supplementation for grazing sheep on growth performance and blood parameters. Our hypothesis was that the supplementation of algae in grazing sheep had no deleterious effects on the weight gain and blood parameters of the animals.

## 2. Materials and Methods

### 2.1. Ethical Statement

This research was carried out following the protocols established by the Professional Committee for the Standardization of Experimental Animals of the Universidad Autonoma del Estado de Mexico (project ID: 4974/2020 CIB).

### 2.2. Grassland and Algae Preparation

A total of two paddocks were used, one control paddock and one treatment paddock. Both were used extensively with continuous grazing at Estancia Josefina, located 70 km north of Punta Arenas, Magallanes, and Chilean Antarctica Region, on Route 255. Each paddock had an area of 20 hectares and 5 sheep per paddock. To determine its botanical composition, the percentage of coverage and presence of species were estimated, using a 50 × 50 cm quadrat, heterogeneously distributing 10 times the quadrant over the entire surface of both paddocks [21], and then cutting of the plant material was carried out in each; then, the fresh plant matter was dried at 60 °C for 48 h for the estimation of DM production of the paddocks. Additionally, the Gs algae were provided by the Algina Chemicals plant, located in the Magallanes and Chilean Antarctica Region. For this, 10 kg of dry matter of Gs algae was sieved with the 4 mm Standard Test Sieve No. 5 (brand W.S. Tyler, Mentor, OH, USA, STATE).

### 2.3. Exp 1. In Vitro Trial

#### 2.3.1. Chemical Composition

Samples of grassland and algae were dried at 60 °C for 48 h and separately pooled and grounded in a hammer mill with a 1 mm screen (Arthur Hill Thomas Corporation, Philadelphia, PA, USA) and analyzed (three replicates) for DM (930.15), CP (Kjeldahl N × 6.25, 990.03), ether extract (EE) (945.16), and ash (967.05) according to Association of official analytical collaboration [22]. The ash-free neutral detergent fiber (NDF), acid detergent fiber (ADF), and lignin content of samples were measured (Fibertec 1010, Tecator, Höganäs, Sweden) according to Van Soest et al. [23]. Mineral content (Ca and P) was determined as described by Peters [24].

#### 2.3.2. In Vitro Gas Production

Rumen fluid samples (approximately 300 g of liquid and 200 g of solids) from three fistulated 5-year-old Dohne Merino ewes [with an average body weight (BW) of 47 ± 1.4 kg (mean ± standard deviation, SD)] were collected, pooled, and homogenized prior to incubation. The ewes were previously fed on natural grassland (control diet). The content from all three animals was pooled and homogenized to ensure a representative microbial inoculum, as recommended by González Ronquillo et al. [25]. The homogenized rumen fluid was then filtered through four layers of cheesecloth to remove large particulate matter and was immediately transferred to the laboratory in a pre-warmed thermos flask under anaerobic conditions for use in in vitro incubations.

In vitro gas production (IVGP) was determined according to Theodorou et al. [26]. In brief, 0.800 g DM of each diet, including (1) 0.800 g of Grassland and (2) 0.560 g of Grassland + 0.240 g Gs (70:30 ratio), was placed in 125 mL glass jars in triplicate (3 flasks per treatment), with three incubation batches using a completely randomized design. Each bottle contained 10 mL of sheep rumen fluid and 90 mL of a buffer solution, and they were incubated in a water batch at 39 °C. The gas pressure was recorded at 3, 6, 9, 12, 24, 36, 48, 60, 72, 84, and 96 h of incubation using a digital pressure gauge (HD2124, Delta OHM, Via Marconi, Caselle Di Selvazzano, Italy). After 96 h of incubation, pH and dry matter disappearance (DMD) were determined. Additionally, the ammonia nitrogen (NH_3_-N) concentration was measured using the phenol hypochlorite method [27]. Pressure readings (PSI) were corrected and converted to gas volume (mL) by performing a linear regression [y, gas volume (mL) = 2.1165 x, x = psi, R^2^ = 0.9979].

To estimate gas production kinetics, data (mL/g DM) were fitted with the NLIN procedure of SAS according to Theodorou et al. [26], using the following model: GP = B (1 − e^−ct^), where GP = gas production (mL gas/g DM); B = total gas production (mL gas/g DM); c = degradation rate compared with the time (hours); and t = time (h). In vitro DMD (IVDMD, mg/100 mg) and organic matter disappearance (IVOMD, mg/100 mg), metabolizable energy (ME), short chain fatty acids (SCFA), and microbial crude protein (MCP) production were also estimated as described by Getachew et al. [28].

After the incubation periods, the bottles were opened, and the contents were filtered and then dried at 60 °C for 48 h, in order to evaluate the loss of dry matter (DMD 96 h). In addition, gas production at 24 h (GY24) was determined by calculating the volume generated (mL of gas/g DM) by dividing the amount of DMD (g), according to González Ronquillo et al. [25].

Fermentation efficiency at 96 h (PF96) was estimated by the ratio of in vitro DMD (mg) to the total volume of gas produced (GP96, mL), following the methodology of Blümmel et al. [29].

#### 2.3.3. Methane (CH_4_) Production

To determine CH_4_ levels, 0.200 g DM samples of each diet were incubated in 100 mL glass syringes in triplicate. This process was repeated in three incubation batches, with 20 mL of a buffer solution and 10 mL of sheep rumen fluid [26]. Gas volume (mL gas/200 mg DM) and CH_4_ were measured after 4, 8, 12, and 24 h of fermentation. For CH_4_ emission, a 1 mL sample of the gas generated in the upper space of each gas handling syringe was taken with the help of a three-step stopcock, and it was diluted 1/100, transferred to another 100 mL glass gas syringe (1100 TLL, Hamilton Company, Reno, NV, USA), and passed through a CH_4_ detector (PHG100, PANGEA, Jiaxing, China) to record results in parts per million (ppm). The average value obtained from each sample was adjusted to the volume of gas produced.

#### 2.3.4. Moisture Retention Test

Water retention (Rh) was determined following Wang et al. [30]. Briefly, 1 g of DM of each diet (in triplicate) was weighed on a Whatman paper filter and placed in a funnel with a flask to collect excess water. An amount of 25 mL of water was added to each sample, and the weight difference was recorded as the initial Rh at 0 h. Subsequently, the weight was measured at 1, 2, 4, 6, 8, 12, 24, and 48 h. The moisture retention capacity (MRC) was determined. Prior to the test, samples were dried at 60 °C for 24 h. The moisture retention capacity was evaluated by calculating the weight in the dry sample (Ra) as a percentage: Ra (%) = (W_n_ − W_0_)/W_0_, where W_0_ and W_n_ represent the weights of the sample before and after the 48 h water test at 20 °C. Wet samples were prepared by adding 25 mL of water to 1000 g of DM, and moisture retention capacity was evaluated by the percentage of residual water in the wet sample (Rh), as follows:

Rh (%) = H_n_/H_0_ × 100,

where H_0_ and H_n_ are the weights of water in the sample before and after introducing the silica gel at 20 °C after, 1, 2, 4, 8, 12, 24, and 48 h of the test.

Rh (%) = [(W_f_ − W_i_)/W_i_] × 100, where W_f_ = weight of final sample + water; W_i_ = weight of initial sample as DM.

### 2.4. Exp 2. In Vivo Trial

#### Experimental Design and Sample Collection

A total of ten 5-year-old Dohne Merino ewes with average body weights (BW) of 47 ± 1.4 kg (mean ± standard deviation, SD) were used for the study. The sheep were not lactating before the experiment, and they were in a stable maintenance state. The trial lasted 31 days and was preceded by an adaptation period of 9 days. The following two dietary treatments (5 animals each) were included: (i) Grassland group (24 h grazing) and (ii) Grassland + Gs group [20 h of stabling for the supplementation of Gs (450 g DM/animal per day) and 4 h (1500–1900 h) of continuous grazing]. The algae ration for each animal per day was estimated from the literature [31,32] in relation to the total DM consumed by the sheep in this assay (1500 g DM/animal per day) [33]. The purpose of using this level was to explore the potential metabolic and physiological effects of Gs in sheep, particularly considering that this is the first known study evaluating Gs in small ruminants. While this level exceeds what might be used in practical ruminant feeding systems, it allows for the clear detection of any biological responses or tolerance limits under controlled conditions, which can inform future dose–response or optimization studies.

For the Grassland + Gs group, once grazing was completed, they were moved daily in a house feeding for the supplementation of Gs. Daily consumption of the seaweed was estimated at 32% of the total seaweed ration.

All animals were weighed following 16 h fasting using a calibrated scale, with the averages BW measured at the initial and final evaluation, the average weight gain of both groups was obtained, and the average daily gain (ADG, g/d) per animal and treatment was adjusted. Body condition score (BCS) was assessed at the beginning (day 0) and at the end of the experimental period (day 31) by palpation of the muscle and adipose mass surrounding the spinous and transverse processes of the lumbar spine on a scale from 1 to 5 (scale of 1 = very lean, 5 = very fat) [34]. Blood samples were collected after overnight fasting through the jugular vein using 10 mL vacutainer tubes [BD^®^ Vacutainer^®^ (Becton, Dickinson and Company, Franklin Lakes, NJ, USA) tube with SST™ II Advance gel Measure 13 × 75 mm, plastic, serum separator gel additive, yellow stopper] on days 0 and 31. Serum samples were obtained by centrifuging the blood tubes for 10 min at 3500× *g* at 10 °C and then were frozen at −20 °C until subsequent analysis. The serum samples were analyzed (three replicates) for glucose (Glu; mg/dL), total protein (TP; g/dL), and albumin (Alb; g/dL) by an autoanalyzer (CS-T24O, Dirui Industrial Co., Ltd., Changchun, China). Globulin (Glo; g/dL) was calculated as the difference between TP and Alb (Glo = TP − ALB). Additionally, the minerals and electrolytes including calcium (Ca; mg/dL), phosphorus (P; mg/dL), magnesium (Mg; mg/dL), sodium (Na; mEq/L), potassium (K; mEq/L), and chlorine (Cl; mEq/L) were determined in an Electrolyte Analyzer (EasyLyte, Medica Corporation, Bedford, MA, USA) according to the manufacturer’s instructions.

### 2.5. Statistical Analysis

In vivo data on BW, ADG, and BCS, as well as in vitro gas production parameters, were analyzed using the general linear model (GLM) procedure of SAS version 9.2 (SAS/STAT, SAS Institute Inc., Cary, NC, USA) using the following model: *y*_ij_ = μ + *T*_i_ + *e*_ij_, where *y*_ij_ is the dependent variable, μ is the overall mean, *T*_i_ is the treatment effect, and *e*_ij_ is the error. One-way analysis of covariance (ANCOVA) was performed for ADG and Final BW considering BW as a covariate. The partial eta squared statistic (η2) was applied, with small, medium, and large effects considered for η2 values of 0.01, 0.06, and 0.14, respectively.

The blood parameters were analyzed based on a completely randomized design (CRD) with two treatments (diets) and five replicates (sheep) by a mixed model of SAS version 9.2 for repeated measurements. The fixed effects in the model were the dietary treatment (diet), the time of sampling (time), and their interaction (diet × time), while sheep was included as a random factor. Least-square means (LSM) were calculated and tested for differences by Tukey’s test. Significance was considered at *p* values ≤ 0.05, and values between 0.05 and 0.10 were considered tendencies.

## 3. Results

### 3.1. Exp 1. In Vitro Trial

#### 3.1.1. Chemical Composition

The percentages of cover for each of the species present in the grassland were *Acaena magellanica* (34.0 ± 10.0%), *Poa* sp. (29.2 ± 10.9%), *Acaena pinnatifida* (5.2 ± 8.1%), *Festuca gracillima* (5.1 ± 6.4%), and to a lesser extent *Trifolium repens* (2.7 ± 4.2%). Table 1 shows the chemical composition of the grassland and the seaweed. The CP content of the grass was 79.5 g/kg DM, while for the seaweed it was 85.6 g/kg DM, as for the EE and NDF content it was higher for the grass than for the seaweed. The Ca and Cl contents were higher for the grass, while the P and Na content were higher for the seaweed.

#### 3.1.2. In Vitro Gas Production

The IVGP parameters are presented in Table 2. The Grassland group showed higher (*p* = 0.026) potential cumulative gas production (A) compared to the Grassland + Gs treatment. Additionally, the lag time was lower in the Grassland + Gs group (*p* = 0.013). The DMD of the Grassland group was 8.3% higher than Grassland + Gs (*p* = 0.071). However, the Grassland + Gs treatment was superior for PF96 (*p* = 0.004) and MCP (*p* = 0.054), compared to the Grassland group. There were no diet effects for the NH_3_-N concentration (*p* = 0.168).

#### 3.1.3. Methane (CH_4_) Emission

The Grassland + Gs sheep showed lower concentrations of CH_4_ (as mL CH_4_/g DM) after 3 h (*p* < 0.01), 6 h (*p* = 0.01), and 12 h (*p* = 0.01) of incubation in vitro compared to the Grassland group (Table 3). CH_4_ levels also tended to decrease with macroalgae supplementation at 9 h (*p* = 0.10) and 24 h (*p* = 0.07).

#### 3.1.4. Moisture Retention Test

The Grassland + Gs group retained 1.46 times more water with respect to Grassland at zero h (*p* < 0.01; Table 4), and this difference always remained constant, observing that, at 48 h Grassland + Gs retained 1.4 times more water (*p* < 0.01). When expressed as a percentage of its own retention volume, Grassland + Gs always retained more water than Grassland, showing 25% more water retention for Grassland + Gs at 24 h compared to Grassland (*p* < 0.01), and this ratio was maintained until 48 h, with 21% more water retention for Grassland + Gs compared to Grassland (*p* < 0.01).

### 3.2. Exp 2. In Vivo Trial

#### 3.2.1. Animal Performance

The animal performance results are given in Table 5. The average initial BW (IBW, *p* > 0.05) and final BW (FBW, *p* = 0.91) of the animals from each treatment had no differences. Similarly, the initial (*p* > 0.05) and final (*p* > 0.05) metabolic BW values of sheep were not altered. No differences (*p* > 0.05) were observed between the Grassland and Grassland + Gs groups for the ADG. However, Grassland treatment showed a higher final BCS than Grassland + Gs at the end of the experiment (*p* < 0.01).

#### 3.2.2. Blood Parameters

There were no diet effects on TP (*p* > 0.05), Ca (*p* > 0.05), P (*p* > 0.05), Na (*p* > 0.05), K (*p* > 0.05), Mg (*p* > 0.05), and Cl (*p* > 0.05) levels in the serum due to the microalgae supplementation (Table 6). However, the concentrations of Glu (*p* < 0.05) and Alb (*p* = 0.01) were lower in the Grassland + Gs group. The Ca:P ratio did not change (*p* < 0.05) by the treatments.

## 4. Discussion

### 4.1. Exp 1. In Vitro Trial

#### 4.1.1. Chemical Composition of Grassland and Macroalgae

Variations in the chemical composition of seaweeds are created by several factors, including species, season of harvest, growth habitat, and environmental conditions. Indeed, growth rate and chemical composition are influenced by factors such as sunlight exposure, salinity levels, sea depth variations, and water currents [36]. The present results on chemical composition of Gs algae is in line with previously published data [37]. The CP, NDF, and Ash content of 12 macroalgae species (namely *A. esculenta*, *A. nodosum*, *F. serratus*, *F. vesiculosus*, *H. elongate*, *L. digitate*, *P. canaliculata*, *S. latissimi*, *C. crispus*, *P. palmate*, *P. umbilicalis*, and *U. lactuca*) had ranges of 44–152, 271–599, and 10.5–33.7 g/kg DM, respectively [18]. It has been well documented [3] that the proximate composition of macroalgae differed in a phylum (*Chlorophyta*, *Rhodophyta*, and *Ochrophyta*) and seasonal manner. It has been also well known [4] that the different habitats of macroalgae have developed adaptations in their photosynthetic process to thrive in specific marine depths, varying solar radiation levels, and the availability of nutrients in their growth environment. Macroalgae proteins have been identified as high-quality proteins, consisting of a significant proportion of essential amino acids (~46% of the total amino acids) [3,18]. A recent systematic review [4] showed that the red algae (the phylum of Gs) had the highest amounts of CP among macroalgae. In this regard, Min et al. [3] reported that the CP content of red seaweed is comparable with that of high-protein plant feeds like soybean meal. The OM and CP contents of Gs in the present study were lower than those reported by Ortiz-Viedma et al. [37]. These variations in the chemical composition may be due to geographical variation and stage of maturation [3,7]. Macroalgae are recognized for their high levels of minerals, surpassing those found in terrestrial plants [18]. Our results showed that the Gs enriched in Na and Mg agrees with the previous reports [37]. Therefore, this alga has the potential to serve as a valuable source of macrominerals for ruminants. However, it is important to note that its high inclusion levels may raise concerns, as they could exceed the maximum tolerable levels of certain minerals such as sodium chloride [3,4]. In this scene, Pandey et al. [18] demonstrated that specific post-harvest processing techniques are required for reduce the contents of critical minerals before their inclusion in the ruminant diets. To sum up, the present results imply that Gs is an interesting potential source of protein feed; however, more detailed research is required to determine its secondary metabolites, amino acid composition, and fatty acid profile to create a better insight into its use in ruminant diets.

#### 4.1.2. In Vitro Gas Production

In the current experiment, the addition of macroalgae was accompanied with lower potential cumulative gas production and lag time. In confirmation, Hidayah et al. [38] demonstrated that marine algae exhibit relatively low ruminal degradability and gas production, primarily due to their high ash content, which reduces the organic matter content. Zitouni et al. [39] reported that the macroalgae contain high levels of minerals and crude protein (CP), contributing more to biomass than gas production. It has also well been established that certain algae are rich in polysaccharides, carrageenans, alginate, fucoidans, agar, ulvans, xylans, laminarin, and florideans starch, which limits nutrient availability for ruminal microbes [40]. Moreover, Gs as a red macroalga, is particularly rich in sulfated galactans—especially carrageenans—which are complex, high-molecular-weight polysaccharides with limited fermentability in the rumen [6,9]. A recent study [41] found that the adverse impact of marine algae on rumen feed degradability was more pronounced when macroalgae were incubated with grass/clover silage. The impacts of algae on IVGP can be attributed, among other factors, to the presence of specific anti-nutritional phenolic compounds, such as phlorotannins [42]. Similar to terrestrial tannins, phlorotannins can bind with proteins and form complexes with them and fiber to a lesser extent and reduce the fermentability of these fractions in rumen [3,43].

In the present study, although Gs showed higher DMD, it produced less cumulative gas compared to the control. This seemingly contradictory result may be due to the presence of bioactive compounds in Gs, such as phlorotannins and sulfated polysaccharides, which can alter fermentation pathways or inhibit specific gas-producing microbes. These compounds may shift fermentation toward microbial biomass synthesis or propionate production, both of which yield less gas [43,44,45,46]. Furthermore, some non-fiber soluble fractions in Gs may be digested or assimilated without substantial gas formation. Therefore, gas volume alone may not fully reflect substrate degradability when fermentation-modulating agents are present, and complementary parameters like microbial protein synthesis and partitioning factor should also be considered.

In the literature, the in vitro fermentation of various seaweeds has been evaluated using a range of inclusion levels. In line with these results, Rjiba-Ktita et al. [7] showed that the gas production, fermentation rate, and DMD were lower for macroalgae (*Chaetomorpha linum* and *Ulva lactuca*) than barley grass. Likewise, Machado et al. [47] showed that all 20 species of macroalgae (seven green, six brown, and four red algae) had lower gas production than decorticated cottonseed meal. Guinguina et al. [43] showed that the total gas production linearly decreased with the inclusion of *B. hamifera* (2.5%, 5.0%, and 7.5% DM) in grass silage. The addition of macroalgae (20% of total DM; *A. nodosum*, *F. vesiculosus*, *F. serratus*, *F. vesiculosus*, and *P. palmata*) to maze silage reduced in vitro gas production [18]. The inclusion of *A. taxiformis* or *Oedogonium* at levels exceeding 10% (DM basis) was associated with lower in vitro OMd of grass hay. However, green (*U. rigida*), red (*G. vermiculophylla*), or brown (*S. latissima*) macroalgae inclusion at 25% DM in a single total mixed ration did not affect gas production, while it enhanced DMD and OMD [45]. In our study, a 30% inclusion level was intentionally chosen to evaluate the fermentation response at a high inclusion level, where bioactive compound effects may be more pronounced. Similarly, in a recent meta-analysis [5], macroalgae generally had no effect on both IVGP and digestibility. Contrasting effects of macroalgae supplementation on gas production have been reported to be linked with several factors, including algae species, basal feed, inclusion levels, and fermentation mothed [45]. It has been well established that the differences between forage and aquatic plants can be attributed not only to the quantity of fiber but also to its structure and constituents [3,7]. Seaweeds, as a source of dietary fiber, possess distinct physicochemical properties compared to terrestrial plants, which can impact ruminal fermentative processes. Seaweeds are rich in sulphated polysaccharides, a component that is absent in terrestrial plants. The biochemical intricacy of algal cell wall composition, potential cellulose association with other polymers, and the varying degree of crystallinity of algal cellulose have been hypothesized to restrict microbial enzyme access to these substrates [3]; however, this assumption requires further confirmation through structural and histological analyses.

#### 4.1.3. Methane (CH_4_)

Reducing methanogenesis can liberate molecular H_2_ for utilization in pathways that result in the production of rumen fermentation end products (e.g., VFA) that can serve as energy source for the host animal [3]. Our results revealed that the CH_4_ levels decreased with macroalgae supplementation because of the reduction of microbial activity. Seaweeds are known to contain over 1500 secondary metabolites and can be rich in halogenated aliphatic organic compounds, such as chlorobromomethane and bromoform [42]. Moreover, species within the *Rhodophyta* family (like Gs) have been characterized by their high and broad-spectrum antimicrobial activity due to their high concentration and diversity of volatile halogenated compounds [46]. Bromoform, a naturally occurring compound in red seaweeds (*Rhodophyta*), is considered the main substance responsible for reducing methane production. It acts by disrupting key microbial processes involved in methanogenesis [47].

The potential of marine algae to mitigate methane emissions has been frequently approved using both in vivo and in vitro approaches [5]. An in vitro study [17] showed that a dose of 5% DM, *A. taxiformis*, and *Z. farlowii* reduced methane production by up to 74% and 11%, respectively. It has also been demonstrated with *B. hamifera* inclusions at 2.5%, 5.0%, and 7.5% of grass silage [43]. Similarly, a recent in vivo study reported that the low levels of red seaweed (*A. taxiformis*; 0.05% to 0.2% OM) in a beef TMR-based diet reduced CH_4_ emissions by up to 98% [48]. Likewise, the addition of *A. taxiformis* (0.25% to 0.20%, DM basis) decreased average daily CH_4_ emission and CH_4_ yield in dairy cows by 65% and 55%, respectively [49]. Min et al. [3] reviewed the existing data, both in vitro and in vivo, associated with the inclusion effects of macroalgae in ruminants, confirming their mitigatory impact on enteric CH_4_ emissions. Additionally, a meta-analysis revealed that the dietary inclusion of macroalgae species decreases CH_4_ production in ruminant animals [5]. The effectiveness of macroalgae in reducing methane production during rumen incubation has been linked to the concentration of halogenated bioactivity including bromoform and di-bromochloromethane [17]. In this sense, Tomkins et al. [50] reported that the organobromine in marine algae inhibited microbial methanogenesis. It has also been well established that, in methanogenesis, metalloenzymes within the Wolfe cycle could be inhibited by bromoform. This cycle is described as a CO_2_ reduction process involved in methane formation using hydrogen [4,51]. Additionally, coenzyme M methyltransferase is competitively bound by bromoform, which suppresses the methyl transfer mechanism essential for methane production [52]. Moreover, Pandey et al. [18] showed that the CH_4_ mitigating properties of macroalgae were associated with a marked reduction in the abundance of the dominant CH_4_ producing archaea (*Methanobrevibacter*, phylum *Euryarchaeota*) in the post-fermentation rumen fluid.

Despite these promising findings, a key limitation of the current study is the lack of data on in vitro fermentation end-products (e.g., VFA profiles) and differential rumen microbial population analysis. Such data would have enabled a clearer understanding of the mechanistic basis for CH_4_ reduction. We strongly recommend that future research integrates VFA quantification and microbial community profiling to better elucidate how macroalgae modulate in vitro ruminal fermentation pathways and microbial ecology. Moreover, the anti-methanogenic potential of macroalgae should be validated in vivo to design optimal strategies for implementation.

#### 4.1.4. Moisture Retention Test

One of the characteristics of algae that should be taken into account when using them in animal feed is their structure, since they are made up of polysaccharides, which form a three-dimensional network, as well as forming polar hydrogen bonds, which favors water encapsulation [53]. In the present study, it was observed that the Grassland + Gs treatment retained up to 25% more water than the Grassland treatment alone, and it remained constant up to 48 h. This water retention capacity could influence the hydration of the animal, increasing its need for drinking water consumption to maintain the water balance and the correct functioning of its organism. According to a study carried out with the inclusion of *M. pyrifera* in the diet, it led to an increase in water consumption and consequently urine excretion [31]. Another point to consider is that most algae are rich in minerals, which could make them a less than ideal food in situations of water scarcity and drought due to the high demand for drinking water needed to excrete minerals from the body [54].

### 4.2. Exp 2. In Vivo Trial

#### 4.2.1. Animal Performance

In the present study, there were no diet effects on the BW and ADG of sheep. Similarly, the addition of macroalgae (*Halimeda opuntia*) powder (1% DM of diet) altered neither BW nor the ADG of growing lambs [55]. It has also been reported that the dietary addition of 10 mg/kg of macroalgae (*A. nodosum*) had no effect on the BW and growth rate of steers [56]. No significant differences were found in the ADG of Angus-Hereford beef steers along with no change in milk yield in Jersey cows fed TMR diets with low levels of *A. taxiformis* (0.25–0.5% DM of diet) and *A. nodosum* (113 g/d), respectively [57,58]. Consistent with our results, Sandvik [59] observed no significant effects of macroalgae inclusion (6% *L. hyperborean*, DM basis) on the DMI and ADG of lambs in a 90 d trial. Ktita et al. [60] have also found that the inclusion of macroalgae (*Ruppia maritima* and *Chaetomorpha linum*) at a level of 20% in barley and soybean meal had no significant effect on the DMI, ADG, nutrient digestibility, and nitrogen balance of lambs. Similarly, the dietary supplementation of 10% (DM basis) seaweed (*A. nodosum* and *Laminaria cloustoni*) for Ayrshire dairy cows did not affect milk yield or fat percentage [61]. However, a recent meta-analysis [62] of a total of 84 articles with beef cattle, sheep, and goat demonstrated that supplementing diets with algae is linked to an inclination for weight gain consistent with the high nutrient content characteristic. Similarly, beef cattle fed diets supplemented with algae have shown positive growth responses, leading to increased weight gains of approximately 2.5% [63]. Additionally, research indicates that providing algal biomass can improve weight gain in heat-stressed cattle [64]. Variability across studies may be explained by factors including animal species, sex, physiological condition, type of algae utilized, and their respective inclusion rates.

Moreover, no effects on ADF and gain-to-feed ratio (G:F) were observed in lambs with the incorporation of algae (at 1% DM) in hay-based diets [65]. Consistently, the lambs fed with *Porphyra* sp. had similar ADG compared to those fed soybean, showing that *Porphyra* sp. can be an alternative high-quality protein for soybean in lamb’s diet [66]. Dietary supplementation of *A. nodosum* at 2% DM of diet resulted in an improved ADG of steers fed a corn-based diet for two 28-day feedlot trials [67]. Kinley et al. [48] found that the ingestion of *Asparagopsis* spp. improved the weight gain of cows without adverse effects on DMI. Similarly, Fike et al. [68] showed that the supplementation of *A. nodosum* extract (at 1.7 to 3.4 kg/ha) led to the enhanced weight gain of lambs fed with endophyte-infected fescue pastures. Therefore, it has been suggested that the presence of biotic or abiotic stressors renders the favorable effects of macroalgae on ruminant growth. A recent systematic review [9] has shown that the positive impacts of dietary macroalgae incorporation on productive performance in ruminants were associated with alga species, inclusion level, and animal growth stage.

#### 4.2.2. Blood Parameters

The blood parameters in both groups were in the normal physiological range for grazing sheep [69]. The present results showed that there were no diet effects on most of the blood parameters. Notably, despite the high sodium (23 g/kg DM) and chloride (98 g/kg DM) content observed in Gs, serum electrolyte levels in the supplemented group remained within normal physiological ranges (Na: 148.1 mEq/L; Cl: 103.1 mEq/L), indicating no evidence of salt toxicity or systemic imbalance. These values align with reference intervals for healthy sheep (Na: 139–152 mEq/L; Cl: 95–113 mEq/L) [70]. No clinical signs of dehydration or reduced intake were observed, suggesting that sheep effectively regulated electrolyte homeostasis during the 31-day trial. However, the elevated mineral content of Gs should be carefully monitored in longer-term applications, particularly in production settings or under limited water access.

However, the concentrations of Glu and Alb were lower in the macroalgae-fed group, which could be related to the low daily forage intake in these animals, as the amount of DM consumed is a key factor for delivering energy to the animal that consequently alters blood Glu levels [59]. It has been shown [71] that the supplementation of macroalgae (*Azolla pinnata*) in a finishing lamb ration did not alter plasma total protein, glucose, cholesterol, triglyceride, albumin, LDL, and HDL metabolites. Additionally, Kannan et al. [72] reported that the dietary addition of seaweed (*S. latifolium*) extract for goats resulted in an increase in blood albumin concentration, which is consistent with our current findings. Unlike our results, the dietary supplementation of 0.25% and 0.50% (DM basis) *Asparagopsis taxiformis* for dairy cows did not affect blood Glu, Alb, and TP in a 28 d experiment [49]. Likewise, blood concentrations of Glu in Jersey cows did not change with feeding brown seaweed (*A. nodosum* at a level of 113 g/d) [57]. The addition of seaweed meal (546 k/kg of *Ascophylum nodosum*) did not change the blood immunity parameters of sheep [73]. The macroalgae supplementation seems to elicit differential glycogenolytic effects in the ruminants. In an earlier study [74] with Boer goats, it was found that seaweed extract supplementation did not have a significant effect on plasma glucose concentration. However, higher plasma Glu concentrations following macroalgae ingestion have been previously reported in goats [75]. More studies are needed to provide further insights about the potential involvement of macroalgae with ruminant blood metabolites, particularly those involved in energy metabolism and liver function.

## 5. Conclusions

The inclusion of Gs at 450 g DM/d in sheep diets did not alter BW, ADG, and blood parameters, with the exception of Glu and Alb. The mitigation of enteric CH_4_ emission was demonstrated under in vitro conditions at this inclusion level and thus should be interpreted as a laboratory finding rather than directly extrapolated to live animal responses. This CH_4_ reduction was accompanied by lower in vitro gas production and IVDMD. Future studies employing longer adaptation periods, larger animal numbers, and varied supplementation strategies are needed to fully evaluate the potential of Gs to reduce CH_4_ emissions without adversely impacting animal health or productivity.

## Figures and Tables

**Table 1:** Chemical composition of the grassland and the *Gigartina skottsbergii* (Gs).

Variable	Ingredient	
	Grassland	Gs
DM, g/kg FM	550	980
OM, g/kg DM	904	722
Ash, g/kg DM	96	278
CP, g/kg DM	80	86
NDF, g/kg DM	488	239
ADF, g/kg DM	289	94
EE, g/kg DM	30	6
NFC, g/kg DM	145	56
Ca, g/kg DM	6	0.05
P, g/kg DM	1	1
Mg, g/kg DM	2	9
Cl, g/kg DM	32	98
Na, g/kg DM	0.50	23.0

DM: dry matter; OM: organic matter; CP: crude protein; NDF: neutral detergent fiber; ADF: acid detergent fiber; NFC: non-fiber carbohydrate, estimated according to the equation: NFC = 100 − (NDF + CP + EE + Ash) [35]; Ca: calcium; P: phosphorus; Mg: magnesium; Na: sodium; Cl: chlorine.

**Table 2:** In vitro gas production and fermentation parameters of the experimental diets.

Variable ^1^	Diets ^2^		SEM ^3^	*p*-Value
	Grassland	Grassland + Gs		
B	148.8 ^a^	131.2 ^b^	3.26	0.03
C	0.03	0.03	0.00	0.54
Lag time	2.50 ^a^	1.10 ^b^	0.25	0.01
Time (h)				
3 h, mL gas/g DM	5.80 ^b^	9.40 ^a^	0.53	0.02
6 h, mL gas/g DM	14.5	18.5	0.92	0.11
9 h, mL gas/g DM	25.3	27.7	2.03	0.45
12 h, mL gas/g DM	37.5	37.3	1.79	0.96
24 h, mL gas/g DM	74.4	67.1	1.64	0.08
36 h, mL gas/g DM	99 ^a^	87.9 ^b^	2.47	0.03
48 h, mL gas/g DM	115.6 ^a^	102.1 ^b^	2.48	0.03
72 h, mL gas/g DM	132.4 ^a^	116.5 ^b^	2.77	0.02
96 h, mL gas/g DM	140.9 ^a^	124.6 ^b^	3.37	0.03
DMD, g/100 g	57.9 ^a^	62.8 ^b^	0.99	0.07
PF96, mL/g DMD 96 h	243.5 ^a^	198.5 ^b^	4.72	0.01
GP24 h, mL/200 mg DM	14.9	13.4	0.33	0.08
ME, MJ/kg DM	11.8	11.4	0.12	0.15
MCP, mg/g DM	546.4 ^b^	598.0 ^a^	9.58	0.05
SCFA, mL/200 mg DM	0.30	0.30	0.00	0.08
NH_3_-N, mg/dL	31.1	26.7	3.33	0.17

^1^ B = total gas production (mL gas/g DM); C = degradation rate compared with the time (hours); DMD = dry matter disappeared at 96 h (g/100 g); PF96 = partition factor (mLgas/g DMD 96 h); GP24h = gas production at 24 h (mL gas/mg DM); ME = metabolizable energy (MJ/kg DM); MCP = microbial protein (mg/g DM); SCFA = short chain fatty acids (mL/200 mg DM). ^2^ P = grassland, P + Gs = Grassland + Gs. Gs = *Gigartina skottsbergii*. ^3^ SEM: Standard error of pooled means. ^ab^ Means within a row with different superscripts differ (*p* ≤ 0.05).

**Table 3:** In vitro methane (CH_4_) production of the experimental diets.

Variable	mL CH_4_/g DM		SEM ^1^	*p*-Value	mL CH_4_/g DM Accumulated		SEM	*p*-Value
	Grassland	Grassland + Gs			Grassland	Grassland + Gs		
3 h	7.2 ^a^	0.4 ^b^	0.35	<0.01	7.2 ^a^	0.4 ^b^	0.35	<0.01
6 h	4.8 ^a^	1.8 ^b^	0.51	0.01	12 ^a^	2.2 ^b^	0.33	<0.01
9 h	8.7	4.1	1.57	0.10	20.7 ^a^	6.3 ^b^	1.78	<0.01
12 h	7.8 ^a^	0.9 ^b^	1.08	0.01	28.5 ^a^	7.2 ^b^	2.78	<0.01
24 h	41.9	28.5	3.92	0.07	70.4 ^a^	35.7 ^b^	1.31	<0.01

^1^ SEM: Standard error of pooled means. ^ab^ Means within a row with different superscripts differ (*p* ≤ 0.05). Gs = *Gigartina skottsbergii.*

**Table 4:** Number of times water retention with respect to initial weight and water retention (%) of experimental diets.

Variable	N Times Retention Water		SEM ^1^	*p*-Value	Rh (%) Water Retention		SEM ^1^	*p*-Value
	Grassland	Grassland + Gs			Grassland	Grassland + Gs		
Time (h)								
0 h	6.0 ^b^	7.5 ^a^	0.05	<0.01	100	100	0.00	>0.05
3 h	5.0 ^b^	6.5 ^a^	0.06	<0.01	82.3 ^b^	87.5 ^a^	0.26	<0.01
6 h	3.5 ^b^	5.7 ^a^	0.25	<0.01	58.8 ^b^	75.7 ^a^	3.65	<0.05
9 h	2.5 ^b^	4.7 ^a^	0.21	<0.01	41.8 ^b^	62.6 ^a^	3.20	<0.05
12 h	1.7 ^b^	4.0 ^a^	0.12	<0.01	29 ^b^	54 ^a^	1.83	<0.01
24 h	0.7 ^b^	3.0	0.04	<0.01	11.9 ^b^	39.7 ^a^	0.49	<0.01
36 h	0.2 ^b^	2.2 ^a^	0.02	<0.01	4.0 ^b^	30.0 ^a^	0.27	<0.01
48 h	−0.02 ^b^	1.6 ^a^	0.02	<0.01	0.0 ^b^	21.8 ^a^	0.204	<0.01

^1^ SEM: Standard error of pooled means. ^ab^ Means within a row with different superscripts differ (*p* ≤ 0.05). Gs = *Gigartina skottsbergii*.

**Table 5:** The performance of grazing sheep fed on experimental diets.

Item	Diets ^1^		SEM ^2^	*p*-Value	Effect Size
	Grassland	Grassland + Gs			
Initial BW, kg	46.6	47.4	1.99	>0.05	0.384
Final BW, kg	48.4	48.1	1.90	>0.05	0.345
ADG, g/d	30.3	25.3	5.29	>0.05	0.332
MBW initial, kg^0.75^	17.1	18.1	0.53	>0.05	0.384
MBW final, kg^0.75^	18.4	18.3	0.66	>0.05	0.345
ADG, g/kg^0.75^	2.3	2.0	0.13	>0.05	0.332
Initial BCS, point	2.6	2.7	0.22	>0.05	0.321
Final BCS, point	3.0 ^a^	2.2 ^b^	0.1	<0.01	0.324

^1^ Diets were as follows: (i) Grassland group: 24 h grazing (*n* = 5); (ii) Grassland + Gs group: 20 h grazing and 4 h of stabling for the supplementation of Gs (450 g DM/animal per day) (*n* = 5); BW: body weight; ADG: average weight gain; MBW: metabolic body weight; BCS: body condition score. Gs = *Gigartina skottsbergii*. Effect size was calculated using the partial eta squared statistic (η2). Small, medium, and large effects are reflected in η2 values equal to 0.01, 0.06, and 0.14, respectively. ^2^ SEM: Standard error of pooled means. ^ab^ Means within a row with different superscripts differ (*p* ≤ 0.05).

**Table 6:** The blood parameters of grazing sheep fed on experimental diets.

Variables	Diet ^1^		Time		SEM ^2^	*p*-Values		
	Grassland	Grassland + Gs	Initial	Final		Diet	Time	Diet × Time
Glu, mg/dL	74.9 ^a^	64.5 ^b^	71.9	67.5	2.89	<0.05	>0.05	>0.05
TP, g/dL	7.5	7.1	7.2	7.4	0.14	>0.056	>0.05	>0.05
Alb, g/dL	3.4 ^a^	3.2 ^b^	3.3	3.4	0.06	0.01	>0.05	>0.05
Glo, g/dL	4.1	3.9	3.9	4.0	0.09	>0.05	>0.05	>0.05
Ca, mg/dL	10.3	10.0	10.4	9.9	0.35	>0.05	>0.05	>0.05
P, mg/dL	5.8	6.2	6.1	5.9	0.64	>0.05	>0.05	>0.05
Na, mEq/L	147.1	147.0	146.0	148.1	0.92	>0.05	>0.05	>0.05
K, mEq/L	5.6	5.2	5.1 ^b^	5.7 ^a^	0.17	>0.05	0.01	>0.05
Mg, mg/dL	2.5	2.2	2.2	2.5	0.15	>0.05	>0.05	>0.05
Cl, mEq/L	100.9	101.7	99.5 ^b^	103.1 ^a^	0.67	>0.05	<0.01	>0.05
Ca/P ratio	2.0	1.7	1.9	1.8	0.21	>0.05	>0.05	>0.05

Glu, glucose; TP, total protein; Glo, globulin; Alb, albumin. ^1^ Diets were as follows: (i) Grassland group: 24 h grazing (*n* = 5); (ii) Grassland + Gs group: 20 h grazing and 4 h of stabling for the supplementation of Gs (450 g DM/animal per day) (*n* = 5). ^2^ SEM: Standard error of pooled means. ^ab^ Means within a row with different superscripts differ (*p* ≤ 0.05).

## Data Availability

The data presented in this study are available on request from the corresponding author (M.G.R.).
